# Entropy and Geometric Objects

**DOI:** 10.3390/e20060453

**Published:** 2018-06-09

**Authors:** Georg J. Schmitz

**Affiliations:** ACCESS e.V., Intzestr. 5, D-52072 Aachen, Germany; G.J.Schmitz@access.rwth-aachen.de; Tel.: +49-241-80-98014

**Keywords:** gradient-entropy, contrast, phase-field models, diffuse interfaces, entropy of geometric objects, Bekenstein-Hawking entropy, Heaviside function, Dirac function, 3D delta function

## Abstract

Different notions of entropy can be identified in different scientific communities: (i) the thermodynamic sense; (ii) the information sense; (iii) the statistical sense; (iv) the disorder sense; and (v) the homogeneity sense. Especially the “disorder sense” and the “homogeneity sense” relate to and require the notion of space and time. One of the few prominent examples relating entropy to both geometry and space is the Bekenstein-Hawking entropy of a Black Hole. Although this was developed for describing a physical object—a black hole—having a mass, a momentum, a temperature, an electrical charge, etc., absolutely no information about this object’s attributes can ultimately be found in the final formulation. In contrast, the Bekenstein-Hawking entropy in its dimensionless form is a positive quantity only comprising geometric attributes such as an area *A*—the area of the event horizon of the black hole, a length *L_P_*—the Planck length, and a factor 1/4. A purely geometric approach to this formulation will be presented here. The approach is based on a continuous 3D extension of the Heaviside function which draws on the phase-field concept of diffuse interfaces. Entropy enters into the local and statistical description of contrast or gradient distributions in the transition region of the extended Heaviside function definition. The structure of the Bekenstein-Hawking formulation is ultimately derived for a geometric sphere based solely on geometric-statistical considerations.

## 1. Introduction

Different senses of entropy [1] lead to five different notions and perceptions of entropy: the thermodynamic, the information, the statistical, the disorder and the homogeneity senses.

Especially the “disorder sense” and the “homogeneity sense” relate to and require the notion of space and time. There is thus a need to introduce explicit spatial information into formulations of entropy. In general, however, the formulations of entropy used in statistical mechanics or thermodynamics (e.g., Gibbs [2,3], Boltzmann [4]) or in information theory (Shannon [5]) do not comprise of any explicit relation to space or time. A prominent example that actually relates entropy to geometry and space is the Bekenstein-Hawking entropy of a Black Hole. Previous derivations of the Bekenstein-Hawking entropy formula [6] are based, for example, on thermodynamics [7], on quantum theory [8], on statistical mechanics of microstates [9] or on information theory [10,11]. All these derivations (to the best of the author’s knowledge) involve the use of physical entities such as temperature and mass. In contrast, the present article limits itself to the use of only geometric/mathematical information and will derive a *formulation revealing a structure that is completely identical* to the dimensionless formulation of the Bekenstein-Hawking entropy of a black hole. It is, however, beyond the scope of this article to discuss the host of possible implications of these findings. 

The formulation of the entropy of a black hole plays an important role in the holographic principle [12,13] and in current entropic-gravity concepts [14,15] describing gravity as an emergent phenomenon. A review of entropy and gravity, which also comprises a section on the entropy of black holes, is found in [16].

Although developed for describing a physical object—a black hole—having a mass, a momentum, a temperature, a charge, etc., absolutely no information about these attributes of this object can ultimately be found in the final formulation. In contrast, the dimensionless form of the Bekenstein-Hawking entropy *S_BH_* is a positive number, which, to obtain the usual form, should be multiplied by Boltzmann’s constant k [17]. The dimensionless formulation, however, only comprises of geometric attributes such as an area *A*—the area of the black hole’s event horizon, a length *L_P_* the Planck length, and a factor of one quarter:

(1) $$S_{BH}=\frac{A}{4L_{p}^{2}}$$

It should thus be possible to construct this formula using a purely geometric approach. Such an approach is attempted in the present article. The approach is based on a continuous 3D extension of the Heaviside function and the phase-field method describing diffuse interfaces.

## 2. A Geometric Object

A 1D object is a line which is confined by a boundary consisting of two points. A 2D object can be defined as an area being confined by a boundary—the periphery—which is a line. A 3D object is a volume which also is confined by a boundary—its surface—which is an area. Any boundary distinguishes the object region of space from the “non-object” region. For an object with dimension n, its boundary has the dimension n–1. Besides these two fundamental characteristics—bulk and boundary—(e.g., volume/surface, area/periphery, length/endpoints) geometric objects have no further physical attributes. In particular, geometric objects do not have attributes like mass, charge, spin and, further, they do not reveal any intrinsic structure. For reason of simplicity and didactics, the following sections will—without limiting the generality of the concept—limit the discussion to the case of a geometric sphere.

## 3. Sharp Interface Description of a Geometric Object

**Figure 1:**
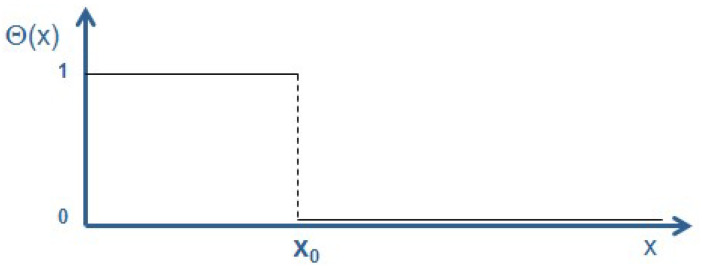
Schematic of the Heaviside function *Θ*(*x*). This function takes the value 1 wherever the object/sphere is present and is 0 elsewhere.

A common way of describing a sphere—or any other geometric object—is to use the Heaviside function *Θ*(*x*) [18], Figure 1:

The volume *V* of a sphere with radius *r*_0_ in spherical coordinates is then given by

(2) $$V=∭\Theta \left(r-r_{0}\right)r^{2}drd\Omega ,$$

where *dΩ* is the differential solid angle:

(3) sinΘdΘdϕ = dΩ.

The Heaviside function delimits any contributions of the integrand larger than *r*_0_ and thus reduces the boundaries of the integral from infinity to *r*_0_:

(4) $$V=4\pi \int_{0}^{\infty }\Theta \left(r-r_{0}\right)r^{2}dr=4\pi \int_{0}^{r_{0}}r^{2}dr\;=\;\frac{4}{3}\pi \;r_{0}^{3}.$$

The surface A of this sphere can be calculated using the gradient of the Heaviside function. Gradients/derivatives of Θ only appear (i.e., have non-zero values) at the positions *r*_0_ of the boundaries of an object. In fact, the definition of the Dirac delta function is actually based on the distributional derivative of the Heaviside function *Θ*(*x*) [18] as

(5) $$\delta \left(x\right):=\frac{d\Theta \left(x\right)}{dx}.$$

Using Equation (5) the surface A of the sphere, as well as the surface of more complex geometric objects, can easily be calculated as follows

(6) $$A=∭\delta \left(r-r_{0}\right)r^{2}drd\Omega =4\pi r_{0}^{2}.$$

This is the first term that is relevant for the entropy of the black-hole—the area of the event horizon—i.e., the boundary making the black-hole distinguishable from the “non-black hole”, or a “sphere” distinguishable from the “non-sphere”. The following sections aim to identify a method of deriving, or at least of providing a reason for the other parameters, i.e., for *L_p_* and ultimately for the factor of one quarter, based on a description of a geometric sphere.

## 4. Phase-Field Description of a Geometric Object

**Figure 2:**
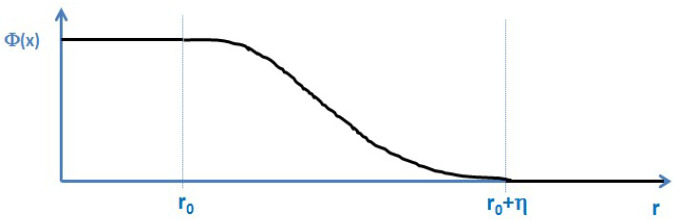
Schematic of the phase-field function *Φ*(*x*). This function takes the value 1 wherever the object/sphere is present and is 0 elsewhere. In contrast to the Heaviside function it reveals a continuous transition over a finite—though very small—interface thickness *η*.

Phase-field models [19,20] developed in recent decades have gained tremendous importance in the area of describing the evolution of complex structures, such as dendrites during phase-transitions. Indeed, they also entered into materials engineering and process design tasks [21]. Similar to the Heaviside function *Θ*, the phase-field *Φ* is a field describing the presence or the absence of an object, Figure 2.

In contrast to the Heaviside function that is based on a mathematically discontinuous transition between the two states “1” and “0”, the phase field approach is based on a continuous transition between these two states within a transition zone width *η*. In case of a very narrow transition width, the phase-field function *Φ*(*x*) can be considered as a continuous, differentiable and 3D formulation of the Heaviside function *Θ*(*x*):

(7) $$\Phi \;\left(r-r_{0}\right)\sim \;\Theta \left(r-r_{0}\right)$$

(8) $$\nabla \Phi \;\left(r-r_{0}\right)\sim \;\nabla \Theta \left(r-r_{0}\right)\;∶=\delta \left(r-r_{0}\right).$$

The gradient symbol without an arrow (*∇*) here has been used to denote the one dimensional derivative in the radial direction. It is distinguished from a three dimensional gradient that is denoted by the *∇* symbol topped by an arrow ($\vec{\nabla }$). Thus the left hand side of the equation is a scalar value while the *δ*-function on the right hand side corresponds to a distribution. A further discussion as to also turning the left-hand side into a distribution is detailed in Section 6.

**Figure 3:**
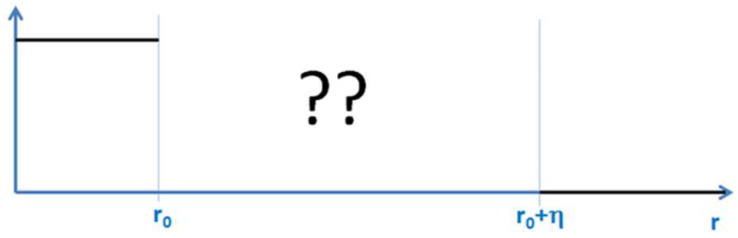
Nothing is known a-priori about the shape of the functions in the small transition zone between the two states. It should also be noted that two state systems have a major importance in quantum mechanical systems and transitions.

The shape of the transition in phase-field models depends on the choice of the potential in the model. A double-well potential, for example, leads to a hyperbolic-tangent profile, while a double obstacle potential leads to a cosine profile of the *Φ*(*x*) function. However, nothing is known a priori, either about the type of potential or about the shape of this function in the transition region in phase-field models, as seen in Figure 3:

## 5. Entropy of Interfaces

General considerations about the shape of the phase-field function in the transition region between 0 and *η* (between *r*_0_ and *r*_0_ + *η*, respectively) require continuity of both Φ(x) and ∇Φ(x) at the transition to the bulk regions, i.e.:

for Φ(x):Φ(0)=1 and Φ(η)=0

for∇Φ(x):∇Φ(0)=0 and ∇Φ(η)=0

for l∇Φ(x):l∇Φ(0)=0 and l∇Φ(η)=0

- $l\;\left(or\;\vec{l}\;in\;3\;dimensions\;\right)$ is a small, non-zero, positive scaling constant having the unit of a length [L]
- $\nabla \Phi \left(x\right)\;\left(or\;\vec{\nabla }\Phi \left(x\right)\;in\;three\;dimensions\;\right)$ has the dimension of an inverse length [L^−1^]
- l∇Φ(x) (in one dimension) and $\vec{l}\vec{\nabla }\Phi \left(x\right)\;$(in three dimensions) *define the contrast* between the two regions. Contrast is a dimensionless, scalar entity and takes values between 0 and 1
- *Φ* also has no physical units. It takes values between 0 and 1.

The “contrast” will play a particularly important role throughout the following sections. From a philosophical/epistemological point of view, “contrast” provides the basis for any type of categorization or classification and thus the basis for any knowledge. From a physical/mathematical point of view, the contrast’s property of being a dimensionless variable seems to be very important since it can therefore enter into the argument of the logarithm.

### 5.1. Discrete Descriptions of the Entropy of an Interface

Entropy has revealed its importance in numerous fields. Some of the most important discoveries are based on entropy; such as (i) the Boltzmann factor in energy levels of systems [4]; (ii) the Gibbs energies of thermodynamic phases [2,3]; (iii) the Shannon entropy in information systems [5]; (iv) the Flory-Huggins polymerization entropy in polymers [22]; and (v) the crystallization entropy in metals [23], to name only some of the major highlights. All these approaches using entropy are based on the well-known logarithmic terms (see e.g., [24]):

(9) $$s=-\overset{N}{\underset{i=0}{\sum }}\Phi_{i}\ln\Phi_{i}.$$

For a two state system (*i* = 0, 1), this formula reduces to

(10) $$s=-\Phi_{0}\ln\Phi_{0}-\Phi_{1}\ln\Phi_{1}.$$

On obeying the constraint of probability conservation:

(11) $$\overset{N}{\underset{i=0}{\sum }}\Phi_{i}=1$$

Equation (10) becomes, for a two states system (*N* = 1)

(12) $$s=-\Phi_{0}\ln\Phi_{0}-\left(1-\Phi_{0}\right)\ln\left(1-\Phi_{0}\right)\;\mathrm{since}\;\Phi_{1}=\left(1-\Phi_{0}\right).$$

As a first step towards the description of the entropy of an interface, different models of crystal growth [25]—the Jackson model, the Kossel crystal, and the Temkin model—will be discussed in detail. Here, the interface between a solid and a liquid serves as an instructive example for any type of transition between two different states.

**Figure 4:**
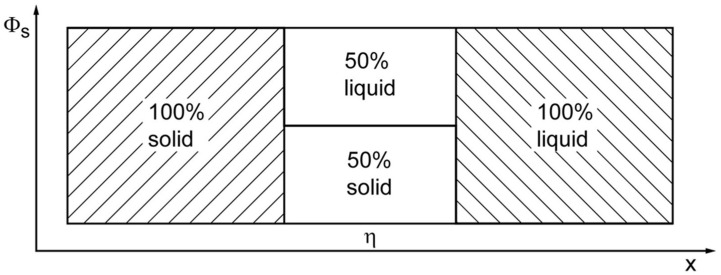
The entropy distribution of the Jackson model generates *Φ* = 0.5 as the most probable value in the interface region (adapted from [26]).

The Jackson model [23] is used to describe the facetted growth of crystals. It assumes an ideal mixing of the two states (solid/liquid) in a single interface layer between the bulk states, Figure 4. The entropy of this interface layer in the Jackson model is described as ideal mixing entropy (see Equations (10) and (12)) which is identical with the Shannon entropy of a binary information system:

(13) $$S=-\Phi \ln\Phi \;-\left(1-\Phi \right)\ln\left(1-\Phi_{0}\right).$$

**Figure 5:**
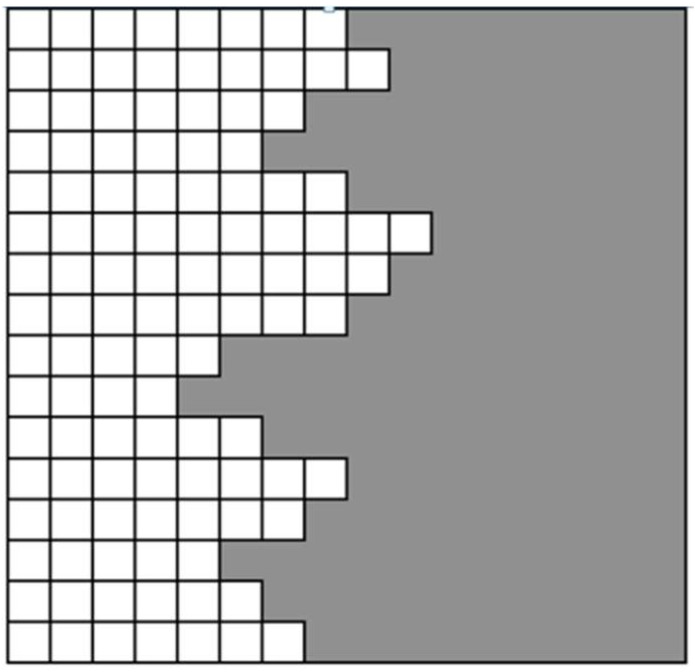
The Kossel model assumes attachment of solid only on the existing solid, i.e., it does not allow for any overhang. Using multiple layers, this model describes a stepwise transition from 100% solid (the four left layers) to 100% liquid (from layer 11 to the right). The projection of layers five to 10 yields a decreasing fraction of solid with increasing layer numbers (adapted from [26]).

The Kossel model (see, e.g., [25]) is a discrete model that is used to describe the growth of crystals with diffuse interfaces, Figure 5. The Kossel model provides the basis for Temkin’s discrete formulation for the entropy of a diffuse interface.

The Temkin model [27] is used to describe growth of crystals with diffuse interfaces. It assumes ideal mixing between two adjacent states/layers in a multilayer interface. The Temkin model describes the entropy of the diffuse interface as:

(14) $$S=-\overset{\infty }{\underset{n=-\infty }{\sum }}(\Phi_{n-1}-\Phi_{n})\ln(\Phi_{n-1}-\Phi_{n}).$$

This model basically allows for an infinite number of interface layers and recovers the Jackson model as a limiting case for a single interface layer. Accordingly, it represents a more general approach.

Highlighting the importance of the Temkin model, one can state that it introduces neighborhood relations between adjacent layers and thus an “order” or a “disorder” sense. Most important, however, it obviously *introduces a gradient and thus a length scale into the formulation of entropy*. The gradient in the Temkin model is identified as follows:

(15) $$d\Phi_{n}=\Phi_{n}\;-\Phi_{n-1}=\overset{nl}{\underset{\left(n-1\right)l}{\int }}\frac{d\Phi }{dr}dr=\;\frac{d\Phi_{n}}{dr}\overset{nl}{\underset{\left(n-1\right)l}{\int }}dr=l\frac{d\Phi_{n}}{dr}\;=l\nabla_{r}^{n}\Phi ,$$

where “*l*” is the distance between two adjacent layers and the gradient is assumed to be constant between these two layers. Actually, Temkin formulated his entropy using the *contrast* between adjacent layers. An extension of the Temkin model to a continuous formulation and to three dimensions is proposed in the next section.

### 5.2. From Discrete to Continuous

**Figure 6:**
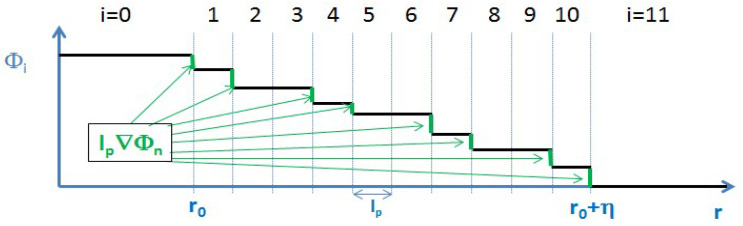
The $\Phi_{n-1}-\Phi_{n}$ values of the Temkin model visualized as contrast, i.e., $l\nabla_{r}^{n}\Phi$ (in green).

Temkin’s discrete formula for the entropy of a diffuse interface, as described in the previous section, can be visualized as follows, Figure 6:

The step to a continuous formulation of Temkin’s entropy, that is already described elsewhere [26], corresponds to assuming an averaged and constant value of the gradient between each pair of cells. Variations of the gradient from cell to cell still remain possible. The number of cells may be infinite and the discretization length l may become extremely small. Some useful relations are:

(16) $$r\left(n\right)=r_{0}+nl\;\mathrm{and}\;dn=\frac{dr}{l}$$

(17) $$S=-\overset{\infty }{\underset{n=-\infty }{\sum }}(\Phi_{n-1}-\Phi_{n})\ln(\Phi_{n-1}-\Phi_{n})=-\overset{\infty }{\underset{n=-\infty }{\sum }}\left\{l\frac{d\Phi \left(nl\right)}{dr}\;\right\}\ln\;\left\{l\frac{d\Phi \left(nl\right)}{dr}\;\right\}.$$

Taking the step from discrete to continuous generates

(18) $$-\overset{\infty }{\underset{n=-\infty }{\sum }}\left\{l\frac{d\Phi \left(nl\right)}{dr}\;\right\}\ln\;\left\{l\frac{d\Phi \left(nl\right)}{dr}\;\right\}\to -\int_{-\infty }^{\infty }\left\{l\frac{d\Phi \left(nl\right)}{dr}\;\right\}\ln\left\{l\frac{d\Phi \left(nl\right)}{dr}\;\right\}dn.$$

Substituting $nl=r-r_{0}\;\mathrm{and}\;dn=\frac{dr}{l}$

(19) $$S=-\int_{-\infty }^{\infty }\left\{l\nabla_{r}\Phi \left(r-r_{0}\right)\right\}\ln\left\{l\nabla_{r}\Phi \;\left(r-r_{0}\right)\right\}\frac{dr}{l}\;.$$

Taking the same steps from one dimension to three dimensions in Cartesian coordinates means (i) extending the radial component product $l\nabla_{r}$ to the full scalar product $\vec{l}\vec{\nabla }\phi$ and (ii) normalizing the other integration directions by some discretization length:

(20) $$S=-\overset{\infty }{\underset{-\infty }{∭}}\left(\vec{l}\vec{\nabla }\phi \right)\ln\left(\vec{l}\vec{\nabla }\phi \right)\frac{dx}{l_{x}}\frac{dy}{l_{y}}\frac{dz}{l_{z}}.$$

Assuming isotropy of the discretization, i.e.,

(21) $$l_{x}=l_{y}=l_{z}=l_{p},$$

ultimately leads to

(22) $$S=-\overset{\infty }{\underset{-\infty }{∭}}\frac{\left(\vec{l}\vec{\nabla }\phi \right)\ln\left(\vec{l}\vec{\nabla }\phi \right)}{l_{p}^{3}}dxdydz.$$

The factor

(23) $$s=\frac{\left(\vec{l}\vec{\nabla }\phi \right)\ln\left(\vec{l}\vec{\nabla }\phi \right)}{l_{p}^{3}}$$

can be interpreted as an entropy density.

Expressed in spherical coordinates Equation (22) yields:

(24) $$\frac{dx}{l_{p}}\frac{dy}{l_{p}}\frac{dz}{l_{p}}=\frac{1}{l_{p}^{3}}r^{2}drsin\Theta d\Theta d\phi =\;\frac{r^{2}}{l_{p}^{2}}\frac{dr}{l_{p}}d\Omega$$

(25) $$S=-∭\left(\vec{l}\vec{\nabla }\phi \right)\ln\left(\vec{l}\vec{\nabla }\phi \right)r^{2}\frac{dr}{l_{p}}\frac{d\Omega }{l_{p}^{2}}.$$

Assuming isotropy (i.e., *Φ* is independent from the angular coordinates), allows one to integrate over the solid angle *dΩ*

(26) $$S=\;-\frac{4}{l_{p}^{2}}\overset{\infty }{\underset{0}{\int }}\left(\vec{l}\vec{\nabla }\phi \left(r-r_{0}\right)\right)\ln\left(\vec{l}\vec{\nabla }\phi \left(r-r_{0}\right)\right)r^{2}\frac{dr}{l_{p}}.$$

Terms with finite, that is non-zero-values of the *∇Φ* yielding contributions to the integral, will only occur at the interface. For very small transition widths *η* of the phase-field *Φ*, proportionality between the terms containing *∇Φ* and the *δ*-function can thus be assumed:

(27) $$\frac{1}{l_{p}}\left(\vec{l}\vec{\nabla }\phi \left(r-r_{0}\right)\right)\ln\left(\vec{l}\vec{\nabla }\phi \left(r-r_{0}\right)\right)\sim \delta \left(r-r_{0}\right).$$

This proportionality can be formulated as an equation by introducing a hitherto unknown constant

(28) $$\frac{1}{l_{p}}\left(\vec{l}\vec{\nabla }\phi \left(r-r_{0}\right)\right)\ln\left(\vec{l}\vec{\nabla }\phi \left(r-r_{0}\right)\right)=constant*\delta \left(r-r_{0}\right).$$

This equation will be further discussed in the following chapter. By preliminarily inserting this relation into Equation (25) yields

(29) $$S=\;-\frac{4\pi }{l_{p}^{2}}\overset{\infty }{\underset{0}{\int }}constant*\delta \left(r-r_{0}\right)r^{2}dr=-constant*\frac{4\pi r_{0}^{2}}{l_{p}^{2}}=-constant*\frac{A}{l_{p}^{2}}.$$

This brings the formulation a step closer to revealing the same structure as the Bekenstein-Hawking entropy. The final step for identifying the factor of one quarter is described in the following section.

## 6. Gradients in Diffuse Interfaces

Considering Φ(r) in the Temkin model highlighted the importance of gradients or contrast for the formulation of the entropy of a diffuse interface. Hitherto, nothing has been specified about the exact shape of $\frac{d\Phi \left(r\right)}{dr}$ or the radial component of the gradient vector in spherical coordinates *∇_r_Φ*.

**Figure 7:**
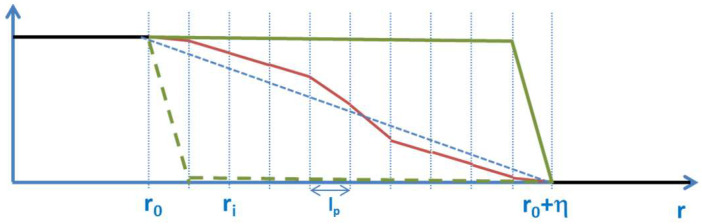
Possible profiles of the Φ function in the transition region. These different shapes lead to different distributions of contrast (see Figure 8).

As a first approximation *∇*_r_*Φ*, could be constant denoting the average gradient between 0 and *η* (see the blue dashed line in Figure 7). The calculation of this *average gradient’s* value in a discrete, spatial formulation is

(30) ⟨∇Φ⟩=∑i=1Nlp∇Φi∑i=1Nlp=1η ,

where the number *N* of the intervals discretizing the interface is defined as

(31) $$N=\;\frac{\eta }{l_{p}}.$$

**Figure 8:**
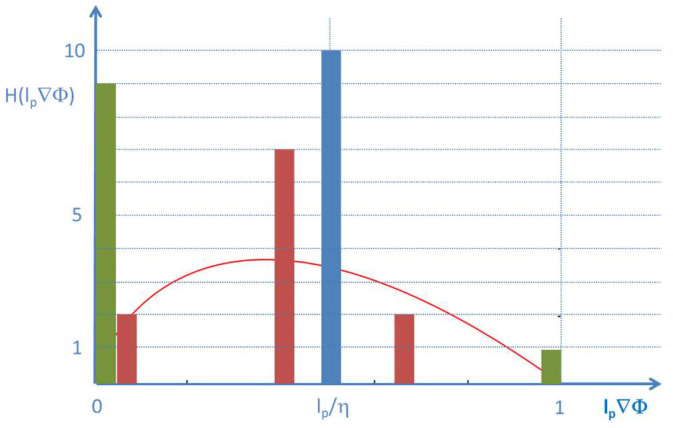
Distributions of contrast in the transition region for the different profiles depicted in Figure 7 : A constant average gradient (blue) leads to an extremely narrow distribution of contrast centered at *l_p_*/*η*. The green shapes lead to high counts for small contrast. The red shape leads to a broad distribution of small and high contrast values. An entropy type distribution of contrast *x_i_* (*N* = 10): H(x) = −10*x*ln(x), is indicated as the red-line overlay.

However, this simple approach does not match the continuity requirements for the gradient at the contact points to the bulk regions. It further leads to a statistically improbable, extremely sharp distribution of the contrast; see the blue bar in the histogram in Figure 8.

The average contrast being calculated from an entropy type *distribution of contrast* reads

(32) $$\langle l_{p}\nabla \Phi \rangle =\frac{\int_{l_{p}\nabla \Phi_{min}}^{l_{p}\nabla \Phi_{max}}\left(l_{p}\nabla \Phi )\ln(l_{p}\nabla \Phi \;\right)d\left(l_{p}\nabla \Phi \right)}{\int_{l_{p}\nabla \Phi_{min}}^{l_{p}\nabla \Phi_{max}}d\left(l_{p}\nabla \Phi \right)}.$$

The minimum gradient in the distribution has the value 0 (or may be finite but very small; see discussion section) while the maximum gradient is 1/*l_p_*. This allows one to fix the boundaries of the integrals to 0 and 1.

(33) $$\langle l_{p}\nabla \Phi \rangle =\frac{\int_{0}^{1}\left(l_{p}\nabla \Phi )\ln(l_{p}\nabla \Phi \;\right)d\left(l_{p}\nabla \Phi \right)}{\int_{0}^{1}d\left(l_{p}\nabla \Phi \right)},$$

This expression, with

(34) $$\int_{0}^{1}d\left(l_{p}\nabla \Phi \right)=1,$$

yields

(35) $$\langle l_{p}\nabla \Phi \rangle =\int_{0}^{1}\left(l_{p}\nabla \Phi )\ln(l_{p}\nabla \Phi \;\right)d\left(l_{p}\nabla \Phi \right).$$

The integral of *x*ln(*x*) gives [28]

(36) $$\int^{\text{​}}x\ln\left(x\right)dx=x^{2}\left[\frac{\ln x}{2}-\frac{1}{4}\right].$$

When integrating over the interval [0, 1], this integral interestingly yields a value of −¼:

(37) $$\overset{1}{\underset{0}{\int }}x\ln\left(x\right)dx=\;1\left[\frac{\ln1}{2}-\frac{1}{4}\right]-0\left[\frac{\ln0}{2}-\frac{1}{4}\right]=-\frac{1}{4}.$$

The *average gradient* or the average contrast resulting from *averaging the distribution* is thus given by

(38) $$\langle l_{p}\nabla \Phi \rangle =-\frac{1}{4}\;or\;\langle \nabla \Phi \rangle =-\frac{1}{4l_{p}}=\;-\frac{1}{4}\frac{1}{l_{p}}=-\frac{1}{4}\nabla \Phi_{max}.$$

Replacing the contrast distribution by its average value, i.e., approximating

(39) $$S=\;-\frac{4\pi }{l_{p}^{2}}\overset{\infty }{\underset{0}{\int }}\left(\vec{l}\vec{\nabla }\Phi \left(r-r_{0}\right)\right)\ln\left(\vec{l}\vec{\nabla }\Phi \left(r-r_{0}\right)\right)r^{2}\frac{dr}{l_{p}}\sim -\frac{4\pi }{l_{p}^{2}}\overset{\infty }{\underset{0}{\int }}\langle \vec{l_{p}}\vec{\nabla }\Phi \left(r-r_{0}\right)\rangle r^{2}\frac{dr}{l_{p}},$$

then yields:

(40) $$S\;\sim \;-\frac{4\pi }{l_{p}^{2}}\overset{\infty }{\underset{0}{\int }}\langle \vec{l_{p}}\vec{\nabla }\Phi \left(r-r_{0}\right)\rangle r^{2}\frac{dr}{l_{p}}$$

(41) $$S=\frac{4\pi }{l_{p}^{2}}\overset{\infty }{\underset{0}{\int }}\frac{1}{4}r^{2}\left|\vec{\nabla_{max}}\Phi \left(r-r_{0}\right)\right|dr.$$

This ultimately leads to

(42) $$S\;\sim \;\frac{4\pi }{l_{p}^{2}}\overset{\infty }{\underset{0}{\int }}\frac{1}{4}r^{2}\delta \left(r-r_{0}\right)dr=\frac{4\pi r_{0}^{2}}{4l_{p}^{2}},$$

and thus to an expression for the entropy of a geometric sphere *S_GS_* revealing the same structure as the Bekenstein-Hawking entropy of a black hole:

(43) $$S_{GS}\sim \frac{A}{4l_{p}^{2}}.$$

## 7. Summary and Discussion

**Figure 9:**
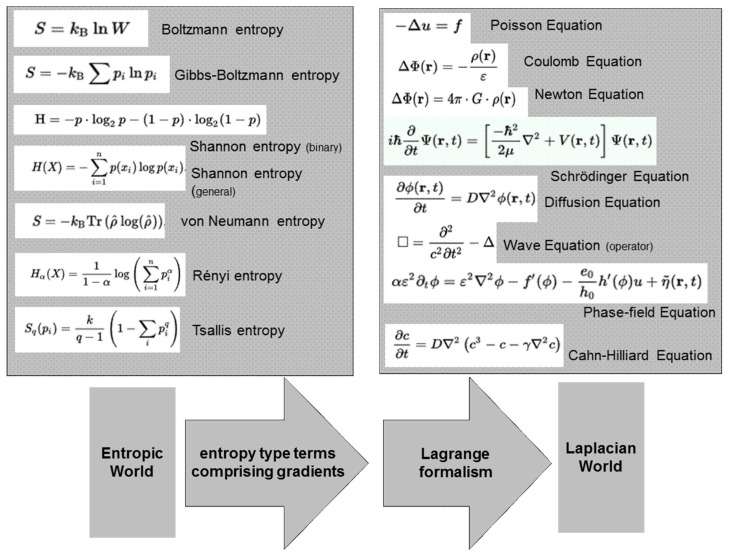
(**Upper left**) Incomplete list of models for a statistical/entropic description of entities in physics and in information theory. Most of these models reveal a logarithmic term as a common ingredient. None of these expressions comprises of gradients and/or Laplacian operators; (**Upper right**) Incomplete list of models for a spatiotemporal description of stationary solutions or for the evolution in physical systems. Many of these models have a Laplacian operator as a common ingredient; (**Bottom**) Entropy formulations comprising gradients, as depicted in the present paper, provide a bridge between these two model worlds.

The structure of the Bekenstein-Hawking formula for the dimensionless entropy of a black hole has been derived for the case of a geometric sphere. This derivation is based only on geometric considerations. The key ingredient to the approach is a statistical description of the transition region in a Heaviside or a phase-field function. For this purpose, gradients are introduced in the form of scalar products into the formulation of entropy based on the Temkin entropy of a diffuse interface. *This introduces a length scale into entropy* and provides a link between the world of entropy type models and the world of Laplacian type models, Figure 9.

The length that is used as the smallest discretization length or as the inverse of the maximum gradient between two states reveals similar characteristics to that of the Planck length.

The minimum gradient—which is set to 0 when making the transition from Equations (28) to (29)—may actually be a finite positive, but non-zero, value. This is calculated as 1/*R_max_* where *R_max_* is some characteristic maximum length over which the transition from 1 to 0 occurs. This *R_max_* might be the radius of the sphere or the radius of the universe outside the sphere. In this case, Equation (33) would contain additional terms leading to minor but perhaps important corrections of the factor of one quarter:

(44) $$\overset{1}{\underset{\frac{l_{p}}{R_{max}}}{\int }}x\ln\left(x\right)dx=-\frac{1}{4}\left(1-{\left(\frac{l_{p}}{R_{max}}\right)}^{2}\right)-{\left(\frac{l_{p}}{R_{max}}\right)}^{2}\ln\frac{l_{p}}{R_{max}}.$$

Such corrections become important (i.e., reach values of a few %) if the ratio of *l_p_*/*R_max_* closely approaches 0.1 and might be subject to further discussions. The major implication of the entropy formulation comprising scalar products or gradients, however, are its prospects of providing a link between entropy type models and Laplacian type model equations as outlined in the final section. 

The major claims of the presented concept are:

1. An entropy can be assigned to any geometric/mathematical object;
2. This entropy is proportional to the surface of the object;
3. This entropy—in the case of a geometrical sphere—has the same structure as the Bekenstein-Hawking entropy.

The entropy of geometrical objects as described in the present article is based on the discretization of the interface between the object and the non-object into a number of microstates. This implies that this interface is not sharp in a mathematical sense but has to have a finite thickness and thus has to be three dimensional (though being extremely thin in one dimension). A mathematically sharp interface, i.e., a 2D description of interfaces—may be an over—abstraction leading to loss of important information.

It is beyond the scope of the present paper to extend the current description and application range of the Heaviside function or to derive equations of gravity. The paper is meant to show (and does so successfully) that the structure of the Bekenstein-Hawking formula can be derived from mere geometric/statistical considerations. All further interpretations and discussions on how to relate this concept to gravity, to thermodynamics, to quantum physics and many other fields of physics, and probably even mathematics, thus require future discussions in a much broader scientific community.

## 8. Outlook

Bridging the gap between statistical/entropy type models and spatiotemporal models of the Laplacian world will lead to interesting physics and to new insights (e.g., on entropic gravity), which may emerge when applying and exploiting the proposed “contrast-concept” in more depth.

A first application of this concept [29] already allowed one to derive the Poisson equation of gravitation including terms that are related to the curvature of space. The formalism further generated terms possibly explaining nonlinear extensions known from modified Newtonian dynamics approaches.
